# 

**Published:** 1917-03

**Authors:** 


PUBLISHED MONTHLY. SUBSCRIPTION $1.00

### ATLANTA JOURNAL-RECORD OF MEDICINE

Successor to { Atlanta Medical and Surgical Journal, Established 1855. and Southern Medical Record, Established 1870. } 63rd Year

Vol. LXIII ATLANTA, GA., MARCH, 1917 No. 12

